# Dr. Eugene Edward Storck

**Published:** 1902-10

**Authors:** 


Dr. Eugene Edward Storck, of Buffalo, died at his residence, 225 East Utica Street, September 9, 1902, aged 47 years. Although his death was due to pulmonary tuberculosis from which he had been ill for some months, the news thereof, nevertheless, came as a sudden shock to all save his very immediate friends.

Dr. Storck received his early educational training in the public and high schools of Buffalo and took his medical degree in 1878 from Buffalo University Medical College.

Dr. Storck belonged to a family of physicians, his father, Dr. Edward Storck, having been a prominent practitioner in

Buffalo for forty years and his son, Edward, 2d, is now a member of the senior class of the medical department, University of Buffalo, having received two years training at Cornell University. In the twenty-four years of his professional career the deceased had acquired a large clientèle, who became his attached friends and to whom his loss is a personal one.

He is survived by a widow, one son, Edward, and a daughter, Eugenia. The funeral was largely attended from the family home, September 12, 1902.

